# Perceptions of health professionals about the implementation of the Adequate and Healthy Eating Promotion Program in a municipality of the Minas Gerais: a qualitative study

**DOI:** 10.1590/S2237-96222025v34e20240408.en

**Published:** 2025-07-11

**Authors:** Juliana Mara Flores Bicalho, Eliete Albano de Azevedo Guimarães, Claudia Di Lorenzo Oliveira, Aline Cristine Souza Lopes

**Affiliations:** 1 Universidade Federal de São João del-Rei, Programa de Pós-graduação em Ciências da Saúde, Divinópolis, MG, Brazil; 2 Universidade Federal de São João del-Rei, Programa de Pós-graduação em Enfermagem, Divinópolis, MG, Brazil; 3 Universidade Federal de Minas Gerais, Departamento de Nutrição, Belo Horizonte, MG, Brazil

**Keywords:** Nutrition Programs and Policies, Food and Nutritional Health Promotion, Diet, Healthy, Food and Nutrition Education, Primary Health Care, Programas y Políticas de Nutrición y Alimentación, Promoción de Salud Alimentaria y Nutricional, Dieta Saludable, Educación Alimentaria y Nutricional, Atención Primaria de Salud

## Abstract

**Objective:**

To analyze the implementation of the Adequate and Healthy Eating Promotion Program in a small municipality in Minas Gerais, from the perspective of health professionals.

**Methods:**

This research used a qualitative approach, with content analysis of semi-structured interviews conducted with professionals from the Family Health Strategy and the multidisciplinary team involved in the implementation of the Adequate and Healthy Eating Promotion Program in Carmo do Cajuru, Minas Gerais. The Program was developed based on the *Instructive Manual: group work methodology for food and nutrition actions in primary health care*, derived from the *Dietary Guidelines for the Brazilian Population*, and lasted approximately five months, with 11 biweekly meetings. The interviews were recorded, transcribed and analyzed thematically.

**Results:**

Twelve health professionals were interviewed. They highlighted the applicability of the program and its positive impact on their daily practice, but reported challenges for its implementation, such as low participant adherence and work overload. Lack of interprofessional engagement was a significant challenge. Facilitating aspects of implementation included the quality of the teaching materials used and the accessible methodology of the Instructive Manual on which the Program was based.

**Conclusion:**

The results indicate that interventions based on these tools had positive impacts, according to the perspective of health professionals, promoting improvements in the quality of information on food and nutrition, as well as in the diet of participants. However, the challenges identified need to be addressed together with health service managers, aiming to increase participant adherence and its effectiveness.

## Resumen

**Objetivo:**

Analizar la implementación del Programa de Promoción de Alimentación Adecuada y Saludable en un pequeño municipio de Minas Gerais, desde la perspectiva de los profesionales de la salud.

**Métodos:**

La investigación utilizó un enfoque cualitativo, con análisis de contenido de entrevistas semiestructuradas realizadas a profesionales de la Estrategia de Salud de la Familia y del equipo multidisciplinario involucrado en la implementación del Programa de Promoción de la Alimentación Adecuada y Saludable en Carmo do Cajuru, Minas Gerais. El Programa se construyó con base en el Manual Instructivo:

**Metodología:**

Metodología de Trabajo en Grupos para Acciones de Alimentación y Nutrición en la Atención Primaria, derivado de la Guía Alimentaria para la Población Brasileña, y tuvo duración aproximada de cinco meses, con 11 encuentros quincenales. Las entrevistas fueron grabadas, transcritas y analizadas temáticamente.

**Resultados:**

Se entrevistaron 12 profesionales de la salud. Destacaron la aplicabilidad del programa y el impacto positivo en su práctica diaria, pero reportaron desafíos para su implementación, como la baja adherencia de los participantes y la sobrecarga de trabajo. La falta de compromiso interprofesional fue un desafío importante. Los aspectos facilitadores de la implementación incluyeron la calidad de los materiales didácticos utilizados y la metodología accesible del Instructivo que basó el Programa.

**Conclusión:**

Los resultados indican que las intervenciones basadas en estas herramientas tuvieron impactos positivos, desde la perspectiva de los profesionales de la salud, promoviendo mejoras en la calidad de la información sobre alimentación y nutrición, así como en la dieta de los participantes. Sin embargo, los desafíos identificados deben abordarse junto con los gerentes, con el objetivo de aumentar la adherencia de los participantes y su efectividad.

**Ethical aspects**

This research respected ethical principles, having obtained the following approval data::

Research Ethics Committee: Universidade Federal de Minas Gerais

Opinion number: 04/1636007

Approval date: 3/7/2019

Certificate of Submission for Ethical Appraisal: 56698716.2.0000.5149

Informed Consent Form: Obtained from all participants prior to data collection.

## Introduction

An adequate and healthy diet is essential for the full development of human beings and for ensuring quality of life (1). Furthermore, it plays a crucial role in prevention and control of chronic non-communicable diseases (NCD), such as diabetes, hypertension and cardiovascular diseases. NCD are the main cause of morbidity and mortality in the world, representing a major challenge for global health and socioeconomic development (2-4). Adopting a diet rich in fruits, vegetables and whole grains and low in ultra-processed foods, added sugars and saturated fats is essential for the health of the population (5).

This scenario requires implementation of public policies that prioritize health education and professional qualification, focusing on promoting adequate and healthy nutrition (6-9), in order to contribute to the food and nutritional security of the population (10) and reduce the risk of NCD (11). Specifically, in Brazil, promoting adequate and healthy nutrition is essential, due to the increasing number of NCD, and it is one of the guidelines of the National Food and Nutrition Policy (1) and the National Health Promotion Policy (12). To implement these policies, the Ministry of Health has been working to create national dietary guidelines, educational materials and health team training (13), especially in Primary Health Care (PHC), due to their role in promoting health and preventing and controlling diseases (14-16). In this context, the *Dietary Guidelines for the Brazilian Population* constitutes an innovative approach.

The second edition of the Dietary Guidelines, published in 2014, constitutes the basis for promotion of healthy and sustainable eating practices. In addition to providing guidance on food choices, it emphasizes the importance of the local social and cultural context of eating practices, as well as the importance of sustainable eating (5). However, for the Dietary Guidelines to be implemented in the daily lives of health professionals, instructional materials need to be produced. In this sense, the *Instructive Manual: group work methodology for food and nutrition actions in primary health care* (17), the first publication derived from the Guidelines, stands out for offering theoretical and practical support for the development of groups to promote adequate and healthy eating in PHC.

The Instructive Manual aims to qualify health professionals to develop collective activities that promote food and nutrition education in a participatory and interactive manner. It is structured in sections that address different aspects of group management with guidelines and suggestions for practical activities and teaching materials. Based on this Instructive Manual, the Adequate and Healthy Eating Promotion Program was developed to support the planning and execution of collective actions by PHC professionals (17). 

This Program aims to contribute to changing professional practices by improving actions to promote adequate and healthy eating (14,15,17,18). However, to ensure its effectiveness, it is essential to evaluate its implementation from the perspective of health professionals, aiming to identify challenges and opportunities in order to optimize work processes and their results (19). Therefore, this study aimed to analyze the perception of health professionals about the implementation of the Adequate and Healthy Eating Promotion Program in a small municipality in Minas Gerais.

## Methods

This article is part of a larger study entitled Evaluation of the Implementation and Effectiveness of the Program to Promote Adequate and Healthy Eating in Primary Health Care, developed in 2019 with the aim of evaluating the implementation and effectiveness of this Program. This article is specifically based on analysis of the perception of health professionals involved in the implementation of the Program in one of the municipalities analyzed in the larger study.

### Design

Qualitative research was conducted using content analysis (20). The qualitative approach is particularly suited to exploring the perceptions and experiences of healthcare professionals, providing in-depth understanding of the challenges and opportunities faced.

### Setting

This research was conducted in Carmo do Cajuru, a municipality in the western macro-region of the state of Minas Gerais, with a population of 23,479 inhabitants (21). The municipality has eight Family Health teams with 100% coverage, in addition to a multidisciplinary team. The Program lasted approximately five months, with 11 biweekly meetings based on the *Instructive Manual: group work methodology for food and nutrition actions in primary health care* (17). The strategies used were: workshops, lasting 40-60 minutes, aimed at promoting dialogue and collective knowledge building; actions in the environment, lasting 15-20 minutes, involving modification of the environment and critical discussion about eating practices; and fixed panels for reflection and communication during the activities.

The following support materials were also made available to health professionals: Logbook; the recipe book entitled *In the kitchen with fruits and vegetables* (22); folders about adequate and healthy eating; and the book entitled *Demystifying questions about food and nutrition* (23).

### Participants

The study included professionals from PHC and the multidisciplinary team who were invited by the Municipal Health Department to implement the Program in the municipality, such as nurses, community health workers, psychologists and a nutritionist. 

Four of the eight professionals forming the municipality’s multidisciplinary team were invited to participate in the implementation of the Program: two psychologists, a nutritionist and a speech therapist. The remainder (physiotherapist, physical education professional, social worker and psychologist) were allocated to the control group to evaluate the effectiveness of the Program. 

The professionals involved in implementing the Program were invited to take part in continuing education conducted by the research group, aiming to qualify them for this purpose. The continuing education lasted 16 hours and was based on the Protocol: Continuing Education for Implementation of Collective Actions to Promote Adequate and Healthy Eating in Primary Care, which was later published by the Ministry of Health (24). The speech therapist participated in the continuing education program, but was not available to participate in the implementation of the Program.

All professionals effectively involved in the implementation of the Program were interviewed. To ensure anonymity, they were identified by their positions, followed by numbers: Community Health Worker 1, Nurse 1, Nutritionist 1, Psychologist 1, and so on.

### Data collection instrument

A script was developed for the semi-structured interviews, aiming to achieve a deeper understanding of both the topic and the personal experiences of the interviewees. To this end, a literature review on the topic was conducted and aspects that could impact the implementation of the Program were discussed. The questions were designed to explore the professionals’ experiences with the Dietary Guidelines and the Instructive Manual, including questions about implementation, participant adherence, challenges and perceptions about the impact of the Program. The script included fields to identify the profile of the professionals, facilitating factors, challenges and suggestions, as well as aspects related to the team, such as planning, integration and communication; and aspects related to service users, such as participation, attendance, interaction and bond with the team.

### Data collection

The interviews were conducted individually by a researcher from the research group with the professionals participating in 2019. They were conducted in a private location, after signing the Free and Informed Consent Form, and were recorded for later transcription and analysis. One of the participants was not available to take part in the interview in person, so that interview was conducted and recorded later on the Skype platform.

### Data analysis

The interviews were transcribed in full by researchers from the research group and analyzed thematically to identify patterns and emerging categories related to the health professionals’ perceptions. The thematic analysis involved three stages: pre-analysis, exploration of the material, and processing the results and interpretation. Thematic analysis allows identification of recurring themes in the participants’ narratives, providing a structured and detailed understanding of the data (25). The categories emerging from the interviews were discussed and refined in meetings with the research team to ensure the validity and reliability of the findings.

## Results

Twelve health professionals involved in implementing the Program were interviewed. The interviews lasted an average of 27 minutes. All participants were women, with an average age of 37 years and an average of six years of experience working in public health. Regarding their positions, five of the participants were community health workers, four were nurses, two were psychologists and one was a nutritionist. Regarding their level of education, four of the participants had completed high school, four had a bachelor’s degree, two had postgraduate specialization and two had master’s degrees.

Based on the analysis of the interviews, three thematic categories were identified: aspects facilitating Program implementation; challenges for implementing the Program; and possibilities for overcoming challenges for implementing the Program. The health professionals valued the Program’s methodology, based on the Dietary Guidelines and the Instructive Manual, highlighting its applicability and positive impact on daily life. However, implementation faced challenges, such as low participant adherence, health professional work overload, and the need for greater involvement of other team members.

Among the main aspects facilitating Program implementation, use of materials such as the Instructive Manual and the recipe book (*In the kitchen with fruits and vegetables*) was cited, as they offered clear and practical guidance. These materials were considered essential for preparing and conducting the activities, as they facilitated communication and provided incentives; they also contributed to the implementation of food and nutrition actions.

The accessible methodology of the Instructive Manual was highlighted for its easy adaptation, allowing various professional categories to participate in leading the groups. Its organized structure and the practical activities suggested in the Instructive Manual also contributed to the implementation of food and nutrition actions.

Other facilitating aspects mentioned included the quality of the information contained in the Instructive Manual (17), which formed the basis for the Program. The interviewees highlighted that the workshops were well planned, providing detailed guidance for each stage, from organization to execution, with lists of materials and suggestions from health professionals for conducting the activities. In addition, the diversity of topics and methodologies helped to avoid monotony and made the meetings more attractive. All of this generated participation and interaction between service users in the actions, which was also highlighted as positive: “Interaction between service users was excellent, they were participative. They did not want [the Program] to end; they wanted it to continue. There was positive feedback from service users regarding improvements in eating habits and even in the quality of sleep” (Community Health Worker 4).

The interviewees also highlighted, as factors that facilitated implementation of the Program, the integration of the PHC team with service users and engagement in the creation of panels, in dissemination of the actions and in active searching for service users, with emphasis on the participation of the Community Health Workers: “There was teamwork with good integration. There was a meeting to divide responsibilities and the actions were planned and organized in advance” (Community Health Worker 5).

Low participant adherence stood out among the main challenges for implementing the Program. Although many showed initial enthusiasm, they faced difficulties in maintaining attendance due to factors such as lack of time, family issues and lack of motivation. There was a decrease in participation over the course of the meetings, attributed to the lack of interest and commitment of service users. Some interviewees associated low participation with the persistence of the biomedical model in health centers, which values ​​individual consultations more than collective activities. The following statement exemplifies this: “Service users prefer consultations. They find difficulty in participating in collective actions to promote health” (Nurse 1).

Another challenge was health professional work overload. Preparing and conducting activities required time and effort, which was added to their other work demands. This aspect was particularly limiting in contexts in which professionals had multiple roles and responsibilities. According to one of the participants: “The main obstacles were workload, lack of time to meet with the team and prepare activities, and actively searching for absent participants” (Nurse 1).

Lack of interest and engagement of some health professionals was also reported as hindering the implementation and integration of actions, compromising the success of the Program. Lack of staff and the absence of some team members made it difficult to plan and implement activities, made worse by workload, preventing face-to-face meetings. In some health centers, for example, activity planning was done via WhatsApp: “There was good integration and communication between the team via WhatsApp. Contact was via WhatsApp” (Psychologist 2).

The difficulty in providing materials for educational activities was another limiting factor: “The materials were easy and cheap in most meetings, but you have to request them from the Health Department well in advance” (Psychologist 1).

Possibilities were identified for overcoming the challenges in implementing the Program, as pointed out by one interviewee: “This methodology allowed service users to be more participative than in other groups, but it requires more time for preparation” (Psychologist 1).

One feature of the Program highlighted by the interviewees was the fact that the workshops can be conducted by different professional categories, not being limited to nutritionists. This facilitates implementation in PHC, allowing different professional categories, including community health workers, to take on the workshops. However, some professionals highlighted the importance of the presence of a nutritionist in certain workshops, especially those that deal specifically with issues related to nutrition: “Other professionals can conduct them, although participants direct questions to the nutritionist in some cases” (Nurse 1).

The nutritionist who supported the planning and implementation of the activities highlighted that the material is clear to conduct: “The proposal provides security in the way of conducting the meetings. The opportunity to learn with material designed to promote adequate and healthy eating in group work is very interesting” (Nutritionist 1).

## Discussion

The results showed a dichotomy between the transformative potential of food and nutrition actions based on the Dietary Guidelines and publications derived from it, such as the Instructive Manual, and the operational challenges faced for their implementation in the daily routine of PHC. 

The importance of the Dietary Guidelines and the Instructive Manual in conducting more participatory collective actions to promote adequate and healthy eating is undeniable, since they encourage educational activities that aim to promote a diet based on unprocessed natural and minimally processed foods, to the detriment of ultra-processed foods, providing a balanced and culturally appropriate diet. Furthermore, the Instructive Manual provides a detailed methodology for conducting groups, including practical workshops and use of interactive teaching materials. However, the effectiveness of interventions depends significantly on the local context and the ability of health professionals to engage and motivate service users. In this sense, low user adherence, work overload and lack of resources were barriers to the implementation of the Program, indicating the need for greater support from health service management (26).

Approximately 20% of individuals attending all meetings adhered to the Program activities. The most frequently cited reasons for non-adherence were insufficient social support and time (27). Low adherence to groups in PHC has been identified in other studies, indicating the need to adopt more effective strategies (28). Low service user participation in collective health promotion actions in PHC may be associated with a curative culture that influences service users and health professionals, limiting the scope of actions. A study on barriers and facilitators for adherence to nutritional intervention in PHC found that barriers included work, self-care, and care for others, while facilitators were related to the structure of the service, the intervention methodology, the building of user-professional bonds, and family support, among others (29).

Health professionals still tend to develop actions based on the biomedical model, prioritizing the understanding of the biological mechanisms of health and disease, and neglecting aspects of people’s daily lives (30). The fragility of the training and qualification processes of health professionals for conducting collective educational activities, with a focus on the emancipation of service users, and the influence of the biomedical model on the organization of health services are relevant aspects (31). In this context, innovative and active methodologies, such as the one covered by this study, can be fundamental for improving collective actions aimed at health promotion.

For activities to be more attractive and sustainable, they need to go beyond “collective consultations” and become dynamic moments that awaken creativity, communication and participation between service users, for a deeper reflection on their experiences (32). In this sense, health professionals observed that the participatory and interactive approach proposed by the Program facilitated the exchange of experiences between participants, which is essential for the success of the interventions.

Another point concerns the need for ongoing training and development of health professionals so that they are prepared to conduct effective food and nutrition interventions. Finally, the importance of institutional support and the provision of adequate resources for implementing interventions stood out. Such support includes time set aside for preparing and conducting groups, as well as materials and appropriate infrastructure, which are essential elements for the success of the actions.

Despite the challenges in implementing the Program, the interventions had a positive impact on the health of service users, with improvements in the quality of their diet, increased consumption of fruits and vegetables, and reduced consumption of ultra-processed foods. In addition, service users reported greater knowledge about nutrition and a better understanding of the importance of healthy food choices. A study that analyzed the effectiveness of the Program in two cities in Minas Gerais found that participants increased the frequency of consumption of natural foods and regular consumption of fruits (27).

Other studies highlight the importance of addressing the promotion of adequate and healthy nutrition in different contexts. For example, the strengthening of these actions for children and adolescents as part of the Health in Schools Program, starting in 2017, in all regions of Brazil (33), as well as the implementation of actions to promote adequate and healthy nutrition in rural areas by community health workers, since Family Health teams in rural areas face different challenges in the work process (34).

In any setting where promotion of adequate and healthy nutrition is proposed, the role of interprofessionality should be highlighted, since the implementation of health promotion programs requires a multifaceted approach, involving the community, health services and decision-makers (35-36). In this sense, the participants pointed out interprofessional collaboration as fundamental for the successful implementation of the Program. This is especially relevant when considering that the adoption of an adequate and healthy diet is a process that takes time and requires maintenance and continuity, whereby longitudinality of the actions poses a challenge for health professionals (24,27,32,35).

In a study conducted on the development of groups to promote adequate and healthy eating with health professionals, the results showed a positive assessment of the group methodology, due to its practical applicability and valuing of service users as autonomous and protagonists of care, in addition to allowing adaptations to various topics, including outside the area of nutrition (31). In this sense, PHC is essential for the expansion of actions, due to its capacity to resolve health problems and its capillarity (37). However, for it to be successful, investment needs to be made in structuring and strengthening PHC, ensuring qualified human resources, adequate infrastructure and quality services. It is also crucial to improve the eating environment where service users live and work, expanding access to healthy food, especially in vulnerable areas. Furthermore, food and nutrition education must be an ongoing and comprehensive process, involving schools, communities and health services (38).

A limitation of this study is that the analysis of the perception of health professionals regarding the implementation of the Program was carried out in only one municipality in Minas Gerais. However, since this is a qualitative study, which involves greater complexity in the analysis of the content of the interviews, we opted for carrying out the study in the municipality of Carmo do Cajuru, due to its peculiar characteristics of being a small municipality, this being predominant in both the state of Minas Gerais and in Brazil as a whole. Although this study was conducted in 2019, it contributes to the literature by providing evidence on the experiences and perceptions of health professionals in the use of the Dietary Guidelines and the Instructive Manual, highlighting the facilitators and challenges of implementing these tools in daily life. The lessons learned can support future interventions to promote adequate and healthy eating in PHC, promoting the health and well-being of the population. However, future studies with more robust designs and in other locations are needed in order to be able to generalize the results and deepen knowledge about the factors that influence the implementation of programs based on the Dietary Guidelines and the Instructive Manual. Furthermore, investment needs to be made in public policies that guarantee access to healthy foods, strengthen PHC, promote food and nutrition education, and encourage production of healthy foods in a sustainable manner, in alignment with the Dietary Guidelines.

This study, conducted in a small municipality in Minas Gerais with PHC professionals, contributes to the body of evidence by discussing the perceptions of health professionals as to the implementation of the Adequate and Healthy Eating Promotion Program, based on the Dietary Guidelines and the Instructive Manual. The results indicate that, despite the challenges, the interventions were positive, according to the perspective of health professionals, promoting improvements in professional qualification and in the nutrition of health service users.

## Data Availability

The highlights of the interviews according to thematic categories used in this research are available in a document sent to the Editor attached to this manuscript.
