# Supplementary material for: Perceptions of health professionals about the implementation of the Adequate and Healthy Eating Promotion Program in a municipality of the Minas Gerais: a qualitative study
Source: Epidemiol Serv Saude. 2025 Jul 11;34:e20240408. doi: 10.1590/S2237-96222025v34e20240408.en (PMC12266651; doi:10.1590/S2237-96222025v34e20240408.en)
Supplement: Translation

# Translation: Percepções de profissionais de saúde na implementação do Programa de Promoção da Alimentação Adequada e Saudável em município de Minas Gerais: pesquisa qualitativa

**DOI:** 10.1590/S2237-96222025v34e20240408.pt

**Authors:** Juliana Mara Flores Bicalho, Eliete Albano de Azevedo Guimarães, Claudia Di Lorenzo Oliveira, Aline Cristine Souza Lopes

**Affiliations:** 1 Universidade Federal de São João del-Rei, Programa de Pós-graduação em Ciências da Saúde, Divinópolis, MG, Brasil; 2 Universidade Federal de São João del-Rei, Programa de Pós-graduação em Enfermagem, Divinópolis, MG, Brasil; 3 Universidade Federal de Minas Gerais, Departamento de Nutrição, Belo Horizonte, MG, Brasil

**Keywords:** Programas e Políticas de Nutrição e Alimentação, Promoção da Saúde Alimentar e Nutricional, Dieta Saudável, Educação Alimentar e Nutricional, Atenção Primária à Saúde

## Abstract

**Objetivo:**

Analisar a implementação do Programa de Promoção da Alimentação Adequada e Saudável em um município de pequeno porte de Minas Gerais, a partir da perspectiva dos profissionais de saúde.

**Métodos:**

A pesquisa utilizou abordagem qualitativa, com análise de conteúdo de entrevistas semiestruturadas realizadas com profissionais da Estratégia Saúde da Família e da equipe multiprofissional envolvidos na implementação do Programa de Promoção da Alimentação Adequada e Saudável em Carmo do Cajuru, Minas Gerais. O Programa foi construído a partir do *Instrutivo*

**Metodologia:**

Metodologia de Trabalho em Grupos para Ações de Alimentação e Nutrição na Atenção Básica, desdobramento do *Guia Alimentar para a População Brasileira*, e teve duração de cerca de cinco meses, e 11 encontros quinzenais. As entrevistas foram gravadas, transcritas e analisadas tematicamente.

**Resultados:**

Foram entrevistados 12 profissionais de saúde. Eles destacaram a aplicabilidade do programa e o impacto positivo na sua prática cotidiana, mas relataram desafios para a sua implementação, como a baixa adesão dos participantes e a sobrecarga de trabalho. A falta de engajamento interprofissional foi um desafio significativo. Aspectos facilitadores da implementação incluíram a qualidade dos materiais didáticos empregados e a metodologia acessível do Instrutivo que baseou o programa.

**Conclusão:**

Os resultados indicam que as intervenções baseadas nessas ferramentas tiveram impactos positivos, segundo a perspectiva dos profissionais de saúde, promovendo melhorias na qualidade das informações sobre alimentação e nutrição, bem como na alimentação dos participantes. Entretanto, os desafios identificados precisam ser tratados em conjunto com os gestores, visando ampliar a adesão dos participantes e sua efetividade.

**Aspectos éticos**

A presente pesquisa respeitou os princípios éticos, obtendo os seguintes dados de aprovação: :

Comitê de ética em pesquisa: Universidade Federal de Minas Gerais

Número do parecer: 04/1636007

Data de aprovação: 3/7/2019

Certificado de apresentação de apreciação ética: 56698716.2.0000.5149

Registro do consentimento livre e esclarecido: Obtido de todos os participantes antes da coleta.

## Introdução

Uma alimentação adequada e saudável é essencial para o desenvolvimento pleno do ser humano e a garantia da qualidade de vida (1). Além disso, desempenha papel crucial na prevenção e controle de doenças crônicas não transmissíveis (DCNT), como diabetes, hipertensão arterial e doenças cardiovasculares. As DCNT são a principal causa de morbimortalidade no mundo, representando grande desafio para a saúde global e para o desenvolvimento socioeconômico (2-4). A adoção de dieta rica em frutas, legumes grãos integrais e pobre em alimentos ultraprocessados, açúcares adicionados e gorduras saturadas é fundamental para a saúde da população (5).

Esse cenário exige a implementação de políticas públicas que priorizem a educação em saúde e a qualificação profissional, com foco na promoção da alimentação adequada e saudável (6-9), de forma a contribuir para a segurança alimentar e nutricional da população (10) e a redução do risco das DCNT (11). Especificamente, no Brasil, a promoção da alimentação adequada e saudável é fundamental, devido ao aumento crescente das DCNT, constituindo uma das diretrizes da Política Nacional de Alimentação e Nutrição (1) e da Política Nacional de Promoção da Saúde (12). Para concretizar essas políticas, o Ministério da Saúde tem trabalhado na criação de diretrizes alimentares nacionais, materiais educativos e qualificação das equipes de saúde (13), especialmente da Atenção Primária à Saúde (APS), devido a seu papel de promoção da saúde, prevenção e controle de agravos (14-16). Nesse contexto, o *Guia Alimentar para a População Brasileira* constitui uma abordagem inovadora.

A segunda edição do Guia, publicada em 2014, constitui a base para a promoção de práticas alimentares saudáveis e sustentáveis. Além de fornecer orientações sobre a escolha de alimentos, enfatiza a importância do contexto social e cultural local das práticas alimentares, e de uma alimentação sustentável (5). Entretanto, para que o Guia seja implementado no cotidiano dos profissionais de saúde, é necessária a produção de materiais instrucionais. Nesse sentido, destaca-se o *Instrutivo Metodologia de Trabalho em Grupos para Ações de Alimentação e Nutrição na Atenção Básica* (17), seu primeiro desdobramento, por ofertar suporte teórico-prático para o desenvolvimento de grupos de promoção da alimentação adequada e saudável na APS. 

O Instrutivo visa qualificar os profissionais de saúde para o desenvolvimento de atividades coletivas que promovam a educação alimentar e nutricional de maneira participativa e interativa. É estruturado em seções que abordam diferentes aspectos da condução de grupos com orientações e sugestões de atividades práticas e materiais didáticos. A partir desse Instrutivo, desenvolveu-se o Programa de Promoção da Alimentação Adequada e Saudável, para apoiar o planejamento e a execução de ações coletivas pelos profissionais da APS (17). 

Esse programa visa contribuir para a mudança das práticas profissionais, qualificando as ações de promoção da alimentação adequada e saudável (14,15,17,18). Entretanto, para garantir a sua efetividade é essencial avaliar a sua implementação, sob a ótica dos profissionais de saúde, visando identificar desafios e oportunidades para otimizar os processos de trabalho e seus resultados (19). Diante disso, este estudo objetivou analisar a percepção dos profissionais de saúde sobre a implementação do Programa de Promoção da Alimentação Adequada e Saudável em município de pequeno porte de Minas Gerais. 

## Métodos

Este artigo faz parte de pesquisa maior intitulada Avaliação da Implantação e da Efetividade do Programa de Promoção da Alimentação Adequada e Saudável na Atenção Primária à Saúde, desenvolvida em 2019 visando avaliar a implementação e a efetividade desse programa. O artigo se baseia especificamente na análise da percepção dos profissionais de saúde envolvidos na implementação do programa em um dos municípios analisados na pesquisa maior. 

### Delineamento

Realizou-se pesquisa qualitativa utilizando análise de conteúdo (20). A abordagem qualitativa é especialmente adequada para explorar as percepções e experiências dos profissionais de saúde, proporcionando compreensão profunda dos desafios e oportunidades enfrentados.

### Contexto

Esta pesquisa foi realizada em Carmo do Cajuru, município da macrorregião Oeste de Minas Gerais, com população de 23.479 habitantes (21). O município possui oito equipes de Saúde da Família com cobertura de 100%, além de uma equipe multiprofissional. O programa teve duração aproximada de cinco meses, com 11 encontros quinzenais baseados no Instrutivo Metodologia de Trabalho em Grupos para Ações de Alimentação e Nutrição na Atenção Básica (17). As estratégias usadas foram: oficinas, com duração de 40-60 minutos, visando promover o diálogo e a construção coletiva do conhecimento; ações no ambiente, com duração de 15-20 minutos, envolvendo a modificação do ambiente e discussão crítica sobre práticas alimentares; e painéis fixos para reflexão e comunicação durante as atividades. 

Foram ainda disponibilizados os seguintes materiais de apoio para os profissionais: Diário de bordo; livro de receitas Na cozinha com as frutas, legumes e verduras (22); fôlderes sobre alimentação adequada e saudável; e livro Desmistificando dúvidas sobre alimentação e nutrição (23).

### Participantes

Participaram da pesquisa profissionais da APS e da equipe multiprofissional que foram convidados pela Secretaria Municipal de Saúde para implementarem o programa no município, como enfermeiros, agentes comunitários de saúde, psicólogos e nutricionista. 

Dos oito profissionais da equipe multiprofissional do município, quatro foram convidados para participar da implementação do programa, sendo dois psicólogos, um nutricionista e um fonoaudiólogo. Os demais (fisioterapeuta, profissional de educação física, assistente social e psicólogo) foram alocados no grupo controle para avaliação da efetividade do programa. 

Os profissionais envolvidos na implementação foram convidados para educação permanente conduzida pelo grupo de pesquisa, visando qualificá-los para tal. A educação permanente teve duração de 16 horas e foi baseada no Protocolo: Educação Permanente para Implantação de Ações Coletivas de Promoção da Alimentação Adequada e Saudável na Atenção Primária, que posteriormente foi publicado pelo Ministério da Saúde (24). A fonoaudióloga participou da educação permanente, mas não teve disponibilidade para participar da implementação do programa. 

Todos os profissionais efetivamente envolvidos na implementação do programa foram entrevistados. Para garantia do anonimato, foram identificados pelos cargos, seguidos de numeração: Agente Comunitário de Saúde 1, Enfermeira 1, Nutricionista 1, Psicóloga 1, e assim por diante.

### Instrumento de coleta de dados

Foi elaborado roteiro para as entrevistas semiestruturadas, visando alcançar um maior aprofundamento da temática e da experiência pessoal dos entrevistados. Para isso, realizou-se revisão da literatura sobre o tema e discutiram-se os aspectos que poderiam impactar a implementação do Programa. As perguntas foram elaboradas para explorar as experiências dos profissionais com o Guia e o Instrutivo, incluindo questões sobre a implementação, adesão dos participantes, desafios e percepções sobre o impacto do programa. O roteiro incluía campos para identificar o perfil dos profissionais, fatores facilitadores, desafios e sugestões, além de aspectos relacionados à equipe, como planejamento, integração e comunicação; e aos usuários, como participação, assiduidade, interação e vínculo com a equipe.

### Coleta de Dados

As entrevistas foram conduzidas, individualmente, por pesquisadora do grupo de pesquisa, com os profissionais participantes em 2019. Foram realizadas em local privado, após a assinatura do Termo de Consentimento Livre e Esclarecido, e foram gravadas para posterior transcrição e análise. Uma das participantes não teve disponibilidade de realizar a entrevista presencial, sendo a entrevista realizada e gravada posteriormente na plataforma Skype.

### Análise dos dados

As entrevistas foram transcritas na íntegra por pesquisadoras do grupo de pesquisa, e analisadas tematicamente para identificar padrões e categorias emergentes relacionadas às percepções dos profissionais. A análise temática incluiu três etapas: pré-análise, exploração do material e tratamento dos resultados e interpretação. A análise temática permite a identificação de temas recorrentes nas narrativas dos participantes, proporcionando uma compreensão estruturada e detalhada dos dados (25). As categorias emergentes das entrevistas foram discutidas e refinadas em reuniões com a equipe de pesquisa, para serem garantidas a validade e a confiabilidade dos achados.

## Resultados

Foram entrevistadas 12 profissionais de saúde envolvidas na implementação do programa. As entrevistas tiveram uma duração média de 27 minutos. Todas as participantes eram mulheres, com média de idade de 37 anos e seis anos de trabalho em saúde pública em média. Quanto ao cargo exercido, cinco das participantes eram agentes comunitários de saúde, quatro enfermeiras, duas psicólogas e uma nutricionista. De acordo com o grau de escolaridade, quatro das participantes concluíram o ensino médio, quatro são graduadas, duas especialistas e duas mestras.

A partir da análise das entrevistas, foram identificadas três categorias temáticas: aspectos facilitadores para implementação do programa; desafios para a implementação do programa; e possibilidades de superação dos desafios para implementação do programa. Os profissionais valorizaram a metodologia do programa, baseado no Guia e no Instrutivo, destacando sua aplicabilidade e impacto positivo no cotidiano. No entanto, a implementação enfrentou desafios, como baixa adesão dos participantes, sobrecarga de trabalho dos profissionais e necessidade de maior envolvimento de outros membros da equipe.

Entre os principais aspectos facilitadores para implementação do programa, foi citado o uso de materiais, como o Instrutivo e o livro de receitas (Na cozinha com as frutas, legumes e verduras), por oferecerem orientações claras e práticas. Esses materiais foram considerados essenciais para a preparação e a condução das atividades, por facilitarem a comunicação e incentivarem; também contribuíram para a implementação das ações de alimentação e nutrição.

A metodologia acessível do Instrutivo foi destaque por sua fácil adaptação, permitindo que diversas categorias profissionais participassem da condução dos grupos. A estrutura organizada e as atividades práticas sugeridas no Instrutivo também contribuíram para a implementação das ações de alimentação e nutrição.

Outros facilitadores mencionados incluíram a qualidade das informações do Instrutivo (17), que basearam o programa. As entrevistadas destacaram que as oficinas foram bem planejadas, fornecendo orientações detalhadas para cada etapa, desde a organização até a execução, com listas de materiais e sugestões de profissionais para condução das atividades. Além disso, a diversidade de temas e metodologias ajudou a evitar a monotonia e tornou os encontros mais atraentes. Tudo isso gerou participação e interação dos usuários nas ações, o que também foi apontado como positivo: “A interação dos usuários foi excelente, eles eram participativos. Não queriam que [o programa] acabasse; eles gostariam que tivesse continuidade. Houve retorno positivo dos usuários sobre melhoria dos hábitos alimentares e inclusive da qualidade do sono” (agente comunitário de saúde 4).

As entrevistadas também ressaltaram, como fatores facilitadores da implementação do programa, a integração da equipe da APS com os usuários e o engajamento na criação dos painéis, na divulgação das ações e na busca ativa dos usuários, com ênfase na participação dos agentes comunitários de saúde: “Houve trabalho em equipe com boa integração. Aconteceu reunião para dividir as atribuições e a ações foram planejadas e organizadas com antecedência” (agente comunitário de saúde 5).

Entre os principais desafios para a implementação do programa, destacou-se a baixa adesão dos participantes. Embora muitos demonstrassem entusiasmo inicial, enfrentaram dificuldades para manter a frequência, devido a fatores como falta de tempo, questões familiares e desmotivação. Houve queda na participação ao longo dos encontros, atribuída à falta de interesse e compromisso dos usuários. Algumas entrevistadas associaram a baixa adesão à persistência do modelo biomédico nas unidades de saúde, que valoriza mais as consultas individuais do que as atividades coletivas. Conforme depoimento: “Os usuários preferem consultas. Há uma dificuldade na adesão com ações coletivas para promoção da saúde” (enfermeira 1).

Outro desafio foi a sobrecarga de trabalho dos profissionais. A preparação e a condução das atividades exigiam tempo e esforço, o que se somava às outras demandas de trabalho. Esse aspecto foi particularmente limitante em contextos nos quais os profissionais acumulavam múltiplas funções e responsabilidades.

De acordo com uma das participantes: “Os principais dificultadores foram a sobrecarga de trabalho, dificuldade de tempo para reunir com equipe e preparar ações e busca ativa dos participantes faltosos” (enfermeira 1).

Também foi relatado o desinteresse e a falta de engajamento de alguns profissionais como dificultadores da implementação e integração das ações, comprometendo o sucesso do programa. A falta de pessoal e a ausência de alguns membros da equipe dificultaram o planejamento e a implementação das atividades, agravadas pela sobrecarga de trabalho, impedindo a realização de reuniões presenciais. Em algumas unidades, por exemplo, o planejamento das atividades foi feito por Whatsapp: “Houve boa integração e comunicação da equipe por Whatsapp. O contato foi por Whatsapp” (psicóloga 2).

A dificuldade na disponibilização de materiais para as atividades educativas foi outro fator limitante: “Os materiais eram fáceis e baratos na maioria dos encontros, porém tem que pedir na secretaria com muita antecedência” (psicóloga 1).

Foram apontadas possibilidades para superar os desafios na implementação do programa, como apontado por uma entrevistada: “Esta metodologia oportunizou que os usuários fossem mais participativos que em outros grupos, porém, demanda mais tempo para preparação” (psicóloga 1).

Uma característica do programa destacada pelas entrevistadas foi o fato de as oficinas poderem ser conduzidas por diferentes categorias profissionais, não se limitando ao nutricionista. Isso facilita a implementação na APS, permitindo que diversas categorias profissionais, incluindo os agentes comunitários de saúde, possam assumir as oficinas. Entretanto, alguns profissionais destacaram a importância da presença do nutricionista em determinadas oficinas, especialmente naquelas que tratam de questões especificamente relacionadas à alimentação: “Outros profissionais podem conduzir, apesar dos participantes direcionarem questões para nutricionista em alguns casos” (enfermeira 1).

A nutricionista que apoiou no planejamento e na implementação das atividades destacou que o material é claro para condução: “A proposta traz segurança na forma de conduzir os encontros. A oportunidade de aprender com um material pensado para promoção da alimentação adequada e saudável em trabalho de grupo é muito interessante” (nutricionista 1). 

## Discussão

Os resultados mostraram uma dicotomia entre o potencial transformador das ações de alimentação e nutrição baseadas no Guia e seus desdobramentos, como o Instrutivo, e os desafios operacionais enfrentados para sua implementação no cotidiano da APS. 

A importância do Guia e do Instrutivo na condução de ações coletivas de promoção da alimentação adequada e saudável mais participativas é inegável, uma vez que incentivam atividades educativas que visam à promoção de uma alimentação baseada em alimentos in natura e minimamente processados, em detrimento dos ultraprocessados, propiciando uma alimentação balanceada e culturalmente apropriada. Ademais, o Instrutivo disponibiliza uma metodologia detalhada para a condução de grupos, incluindo oficinas práticas e uso de materiais didáticos interativos. No entanto, a efetividade das intervenções depende significativamente do contexto local e da capacidade dos profissionais de saúde de engajar e motivar os usuários. Nesse sentido, a baixa adesão dos usuários, a sobrecarga de trabalho e a falta de recursos foram barreiras para a implementação do programa, denotando a necessidade de maior apoio da gestão (26).

 A adesão às atividades do programa foi de aproximadamente 20% dos indivíduos presentes em todos os encontros. Entre os motivos para não adesão, os mais citados foram o apoio social e o tempo insuficientes (27). A baixa adesão aos grupos na APS tem sido identificada em outros estudos, apontando para a necessidade de adotar estratégias mais efetivas (28). A baixa participação dos usuários nas ações coletivas de promoção da saúde na APS pode estar associada a uma cultura curativista, que influencia usuários e profissionais de saúde, limitando o alcance das ações. Estudo realizado sobre as barreiras e facilitadores para adesão à intervenção nutricional na APS encontrou que as barreiras incluíam trabalho, autocuidado e cuidado com o outro, enquanto os facilitadores foram relacionados à estrutura do serviço, à metodologia da intervenção, à construção de vínculo usuários-profissionais, ao apoio familiar, entre outros (29). 

Os profissionais ainda tendem a desenvolver ações baseadas no modelo biomédico, priorizando a compreensão dos mecanismos biológicos da saúde e da doença, e negligenciando aspectos do cotidiano das pessoas (30). A fragilidade dos processos de formação e qualificação dos profissionais para a condução de atividades educativas coletivas, com foco na emancipação dos usuários, e a influência do modelo biomédico na organização dos serviços de saúde são aspectos relevantes (31). Nesse contexto, metodologias inovadoras e ativas, como a deste estudo, podem ser fundamentais para aprimorar as ações coletivas voltadas à promoção da saúde.

Para que as atividades sejam mais atrativas e sustentáveis, precisam ir além de “consultas coletivas”, e se tornar momentos dinâmicos, que despertem a criatividade, a comunicação e a participação dos usuários, para uma reflexão mais profunda sobre suas experiências (32). Nesse sentido, os profissionais observaram que a abordagem participativa e interativa proposta pelo programa facilitou a troca de experiências entre os participantes, sendo essencial para o sucesso das intervenções. 

Outro ponto refere-se à necessidade de qualificação e desenvolvimento contínuos dos profissionais, a fim de que estejam preparados para conduzir intervenções de alimentação e nutrição efetivas. Por fim, destacou-se a importância do apoio institucional e da disponibilização de recursos adequados para implementação das intervenções. Esse suporte inclui tempo reservado para a preparação e condução dos grupos, além de materiais e infraestrutura apropriada, elementos fundamentais para o sucesso das ações.

Apesar dos desafios para implementação do programa, as intervenções impactaram positivamente a saúde dos usuários, com melhorias na qualidade da alimentação, aumento do consumo de frutas e vegetais e redução do consumo de alimentos ultraprocessados. Além disso, os usuários relataram maior conhecimento sobre nutrição e uma melhor compreensão da importância de escolhas alimentares saudáveis. Estudo que analisou a efetividade do programa em dois municípios de Minas Gerais verificou que os participantes aumentaram a frequência de consumo de alimentos in natura e o consumo regular de frutas (27). 

Outros estudos trazem a importância da abordagem da promoção da alimentação adequada e saudável em diferentes contextos. Como exemplo, o fortalecimento dessas ações para crianças e adolescentes como parte do Programa Saúde na Escola, a partir de 2017, em todas as regiões do Brasil (33), assim como a realização de ações de promoção da alimentação adequada e saudável no campo por agentes comunitários de saúde, uma vez que as equipes de Saúde da Família do campo enfrentam diferentes desafios no processo de trabalho (34).

Em qualquer ambiente no qual se propõe a promoção da alimentação adequada e saudável, deve ser destacado o papel da interprofissionalidade, uma vez que a implementação de programas de promoção da saúde requer uma abordagem multifacetada, envolvendo comunidade, serviços de saúde e tomadores de decisão (35-36). Nesse sentido, as participantes apontaram a colaboração interprofissional como fundamental para a implementação bem-sucedida do programa. Isso é especialmente relevante ao se considerar que a adoção de uma alimentação adequada e saudável é um processo que demanda tempo e exige manutenção e continuidade, constituindo a longitudinalidade das ações um desafio para os profissionais de saúde (24, 27,^32^,^35^).

Em pesquisa realizada sobre o desenvolvimento de grupos para promoção da alimentação adequada e saudável com profissionais, os resultados trouxeram uma avaliação positiva em relação à metodologia de grupo, devido à sua aplicabilidade prática e valorização do usuário como autônomo e protagonista do cuidado, além de permitir adaptações para diversas temáticas, inclusive fora da área da nutrição (31). Nesse sentido, a APS é fundamental para a expansão das ações, por sua capacidade de resolutividade dos problemas de saúde e por sua capilaridade (37). No entanto, para que seja exitoso, é necessário investir na estruturação e fortalecimento da APS, garantindo recursos humanos qualificados, infraestrutura adequada e serviços de qualidade. Também é crucial melhorar o ambiente alimentar onde os usuários vivem e trabalham, ampliando o acesso a alimentos saudáveis, especialmente nas áreas vulneráveis. Ademais, a educação alimentar e nutricional deve ser um processo contínuo e abrangente, envolvendo escolas, comunidades e serviços de saúde (38). 

Como limitação deste estudo, aponta-se a análise da percepção dos profissionais de saúde sobre a implementação do programa ter sido realizada em apenas um município de Minas Gerais. No entanto, por se realizar uma pesquisa qualitativa, que envolve maior complexidade na análise do conteúdo das entrevistas, optou-se pela realização do estudo no município de Carmo do Cajuru, por suas características peculiares de pequeno porte, tipo predominante no estado e no país. Ainda que este estudo tenha sido realizado em 2019, contribui para a literatura, ao fornecer evidências sobre as experiências e percepções dos profissionais de saúde na utilização do Guia e do Instrutivo, destacando os facilitadores e desafios da implementação dessas ferramentas no cotidiano. As lições aprendidas podem subsidiar futuras intervenções de promoção da alimentação adequada e saudável na APS, promovendo a saúde e o bem-estar da população. Entretanto, estudos futuros com delineamentos mais robustos e em outras localidades são necessários para generalizar os resultados e aprofundar o conhecimento sobre os fatores que influenciam a implementação de programas baseados no Guia e no Instrutivo. Ademais, é preciso investir em políticas públicas que garantam o acesso a alimentos saudáveis, que fortaleçam a APS, promovam a educação alimentar e nutricional, e estimulem a produção de alimentos saudáveis de forma sustentável, em alinhamento com as diretrizes do Guia.

Este estudo, realizado em município de pequeno porte de Minas Gerais com profissionais da APS, contribui para o corpo de evidências, ao discutir as percepções dos profissionais de saúde na implementação do Programa de Promoção da Alimentação Adequada e Saudável, baseado no Guia e no Instrutivo. Os resultados indicam que, apesar dos desafios, as intervenções foram positivas, segundo a perspectiva dos profissionais de saúde, promovendo melhorias na qualificação profissional e na alimentação dos usuários.

## Data Availability

Os destaques das entrevistas por categorias temáticas utilizados na pesquisa estão disponíveis em documento anexo enviado ao Editor.
